# Validation of the Arabic Severe Respiratory Insufficiency Questionnaire

**DOI:** 10.1186/s12890-021-01644-x

**Published:** 2021-10-11

**Authors:** Marwan F. Alawieh, Rania N. Bzeih, Mohamad F. El-Khatib, Abla M. Sibai, Lilian A. Ghandour, Salah M. Zeineldine

**Affiliations:** 1 Department of Anesthesiology/Inhalation Therapy Department, American University of Beirut Medical Center, Beirut, Lebanon; 2 Nursing Department, American University of Beirut Medical Center, Beirut, Lebanon; 3 Department of Epidemiology and Population Health, Faculty of Health Sciences, American University of Beirut, Beirut, Lebanon; 4 Internal Medicine - Pulmonary Division, American University of Beirut Medical Center (AUBMC), PO Box: 11-0236, Riad El Solh, Beirut, 1107 2020 Lebanon

**Keywords:** Health-related quality of life, Home mechanical ventilation, Respiratory failure, Validation

## Abstract

**Background/objectives:**

Assessment of Health-Related Quality of Life (HRQL) in patients with chronic respiratory insufficiency requiring Home Mechanical Ventilation (HMV) requires a valid measurement tool. The Severe Respiratory Insufficiency (SRI) questionnaire, originally developed in German, has been translated into different languages and tested in different contexts, but has so far not been in use in Arabic-speaking populations. The objective of this study is to validate the Arabic version of the SRI questionnaire in a sample of Arabic-speaking patients from Lebanon.

**Methods:**

Following forward/backward translations, the finalized Arabic version was administered to 149 patients (53 males–96 females, age 69.80 ± 10 years) receiving HMV. Patients were recruited from outpatient clinics and visited at home. The Arabic SRI and the 36-Item Short-Form Health Survey (SF-36) were administered, in addition to questions on sociodemographics and medical history. Exploratory Factor Analysis (EFA) was used to explore dimensionality; internal consistency reliability of the unidimensional scale and its subscales was assessed using Cronbach’s alpha. External nomological validity was examined by assessing the correlation between the SRI and SF-36 scores.

**Results:**

The 49-item Arabic SRI scale showed a high internal consistency reliability (Cronbach alpha for the total scale was 0.897 and ranged between 0.73 and 0.87 for all subscales). Correlations between the SF-36-Mental Health Component MHC and SF-36-Physical Health Component with SRI-Summary Scale were 0.57 and 0.66, respectively, with higher correlations observed between the SF-36 and specific sub-scales such as the Physical Functioning and the Social Functioning subscales [*r* = 0.81 and *r* = 0.74 (*P* < 0.01), respectively].

**Conclusion and recommendations:**

The Arabic SRI is a reliable and valid tool for assessing HRQL in patients with chronic respiratory insufficiency receiving home mechanical ventilation.

**Supplementary Information:**

The online version contains supplementary material available at 10.1186/s12890-021-01644-x.

## Introduction

Health-related quality of life (HRQL), which is comprised of various health components related to functional capabilities, physical conditions, psychological well-being, and social functioning, is a key component of evaluating outcomes of clinical practice and medical interventions [1, 2]. Several tools have been developed and used to assess HRQL, particularly for patients with chronic conditions, including the Sickness Impact Profile [3] and the 36-Item Short-Form Health Survey (SF-36) [4]. These tools, however, examine the general health status regardless of the underlying disease. In the field of pulmonary medicine, disease-specific scales have been developed and validated, and these include the Chronic Respiratory Disease Questionnaire [5] and the St. George’s Respiratory Questionnaire [6] both of which assess fastidious issues related to patients with chronic obstructive pulmonary disease (COPD).

Patients with chronic respiratory insufficiencies, whether caused by obstructive (i.e. COPD) or restrictive ventilatory disorders (i.e. neuromuscular diseases (NMD), restrictive chest wall disorders (RWCD), lung fibrosis, or obesity hypoventilation syndrome (OHS)) are of special medical concern as they represent a unique group given the severe limitations and comorbid effects of their respiratory health. One main intervention for these patients is non-invasive home mechanical ventilation (HMV) that is mostly delivered via a nasal or facial mask. Despite HMV’s proven benefits in improving clinical and physiological parameters specific to patients with severe respiratory insufficiency [7–12], the continued monitoring of the quality of life of these patients remains crucial to assess the impact of disease progression or effect of treatment regimen on daily life [13]. Evaluation of their quality of life has been typically done using the aforementioned generic and other disease-specific scales [10, 14–16]. Yet, these scales lack proper assessment of the important characteristics and specific symptoms exclusive to this patient population leading to an imperfect evaluation of their quality of life [14, 17]. For this reason, the Severe Respiratory Insufficiency (SRI) questionnaire was developed by German researchers to assess central aspects relevant to this patient population [17]. The original SRI, a multidimensional HRQL tool with high reliability [17], has been translated into different languages (e.g. English, Spanish, Norway, Japanese, Chinese, Greek, Hungarian, Portuguese, and French) and tested in different contexts [18–23, 26–28], but notably not in any Arabic-speaking populations of the Arab world. It is worth noting that Arabic is the official language in 27 countries that constitute the Arab League, and the co-official language in 6 additional states [29, 30]. The aim of the current study is to validate an Arabic version of the SRI among patients with chronic respiratory insufficiency.

## Methods

### Patients and study design

Following ethical approval obtained from the Institutional Review Board at the American University of Beirut, a convenient sample of 149 adult patients with chronic respiratory insufficiency receiving HMV was identified and recruited on a rolling basis from clinics specialized in pulmonary medicine until the minimum desired sample size was met based on a 3 subjects per variable recommendation [31, 32]. Patients were recruited across all of Lebanon between March 2016 and May 2018. All patients receiving HMV and clinically stable for more than one month before enrolment were eligible for the study. Patients with a tracheostomy tube, a history of left-sided congestive failure and an exacerbation during the preceding month were not eligible to participate. Eligible patients were initially approached by their attending physicians who explained the objectives of the study and took their approval to be contacted by the research team. Patients were then contacted over the phone by the research team for informed oral consent to participate in the study and for eligibility screening. Consenting eligible patients were visited in their homes for face-to-face interviews.

### Measures

#### SRI scale

The SRI scale is a multidimensional instrument comprised of 49 items, and seven subscales including: Respiratory Complaints (SRI-RC), Physical Functioning (SRI-PF), Attendant Symptoms and Sleep (SRI-AS), Social Relationships (SRI-SR), Psychologic Well-being (SRI-WB), Anxiety (SRI-AX) and Social Functioning (SRI-SF). The SRI questionnaire assesses the patients’ quality of life during the preceding one week based on his/her level of agreement rated using a five Likert-scale from “strongly agree” to “strongly disagree”. Typically, subscale scores are generated or a total scale score is generated (SRI-SS) yielding a score ranging between 0 and 100, with higher scores indicating a better quality of life.

#### SF36

The Arabic SF-36 item scale was administered to measure the general quality of life among our sample. The SF‐36 consists of eight subscales measuring diverse components of health status with a score ranging between 0 and 100; with higher scores indicating better health. The eight subscales are the following: SF‐36‐PF = Physical Functioning; SF‐36‐RP = Role Physical; SF‐36‐BP = Bodily Pain; SF‐36‐GH = General Health; SF‐36‐VT = Vitality; SF‐36‐SF = Social Functioning; SF‐36‐RE = Role‐Emotional; SF‐36‐MH = Mental Health [4].

#### Sociodemographic and other correlates

Sociodemographic data collected included sex, age, highest educational level, occupation, as well as living situation (e.g. who lives with you at home, and who cares for you at home). Questions on cigarette and waterpipe smoking behavior were also included including questions on current smoking status, age at smoking initiation and quitting (for ex-smokers), and quantity smoked per day [allowing the calculation of pack-years]. Data on the underlying diagnosis leading to respiratory failure as well as the main indication for the initiation of HMV was also collected. Finally, the patient was asked to specify the time in years s/he have been using the therapy at home and the hours of use during the day (Additional file 1).

### Cross cultural adaptation

The main purpose behind cross-cultural adaptation is to come up with a comparable version between the original scale (German) and the target version (Arabic).

#### Translation and back translation

The German original was translated to formal Arabic by two different professional sworn translators, whose native language was Arabic. Four authors (M.A., L.G., A.S., and M.K.) reviewed and discussed the two translations, synthesized the results of the two translations and agreed on a common modified Arabic version. The two translations were very similar except for a few minor linguistic differences. The pre-final Arabic version was then back translated to German by a third independent translator, blinded to the original German version; the back-translated version was compared with the original version by a fourth translator. This step was undertaken to ensure correctness of the forward translations.

#### Pretesting the pre-final Arabic version

The version was then pretested among 10 patients receiving HMV, identified and recruited in a similar manner through which the study sample was assembled. The objective of this step was to ensure that the adapted version retains the adequacy of content, clarity of wording and usefulness. No amendments were done as the participants highlighted that the Arabic SRI was easy and understandable. The final Arabic version resulting from the pretest was used within the data collection tools for the validation study.

## Analysis

*Descriptive statistics* were generated for the patients’ socio-demographics, disease characteristics, HMV utilization, general quality of life scores, and SRI scores. *Exploratory Factor Analysis (EFA)* was used to assess the dimensionality of the items in the scale, or its factor structure. *Principal Components Analysis (PCA)* was used as the extraction method, and the scree test to determine the number of factors [33, 34]. The internal consistency reliability of the Arabic-SRI scale and its subscales were assessed by calculating the Cronbach alpha coefficient for the total scale and the subscales. Values of Cronbach’s alpha above 0.7 were considered acceptable [24]. The internal construct validity was assessed using Confirmatory Factor Analysis (CFA) assuming the factor structure as per the original SRI. The model fit was evaluated using the Root Mean Square Error of Approximation (RMSEA) and the Comparative Fit Index (CFI). Values of RMSEA less than or equal to 0.05 indicate a good fit, between 0.05 and 0.08 indicate an adequate fit, and values greater than 0.10 indicate a poor fit. A lower bound of RMSEA 90% confidence interval (CI) less than 0.05, as well as an upper limit less than 0.08, indicate a good fit, whereas a maximum upper bound of 0.10 indicates an acceptable fit. Values of CFI ≥ 0.95 indicate a good fit, values less than 0.95 but greater than 0.90 indicate an adequate fit, and values ≤ 0.90 indicate a poor fit [25]. External nomological validity was finally assessed by examining the Pearson correlation between the Arabic-SRI and the ArabicSF-36. All statistical analyses were done using the Statistical Package for Social Sciences (SPSS) version 23.0.

## Results

### Patients profiles

The questionnaire was surveyor-assisted and had 100% item response rate. However, forty-eight percent of patients did not respond to the item 31 “My marriage/relationship is suffering because of my illness” since they were not married or in a relation at time of filling the questionnaire. Given that the item 31 is one question in determining the social functioning of a patient, it is believed that the response to that item would be in same direction with the entire SRI-SF subscale. Therefore, the “not applicable” answers of the item 31 were substituted by the SRI-SF mean of the same patient calculated based on the responses of the remaining 7 items of that particular subscale. The interview took 15 ± 4 min to complete. The sample was two-thirds female, aged 69.80 ± 10 years, and the majority suffered from COPD (77.00%, n = 114); the remaining patients suffered from a variety of conditions including neuromuscular diseases, Obesity Hypoventilation Syndrome, Restrictive Chest Wall Disorders, among others. About one in four (21.00%, n = 31) were current smokers, two-thirds (62.00%, n = 93) had quit smoking, and 17% (n = 25.00) had never smoked. HMV had been utilized for a mean of 32.60 ± 36.2 months and was applied for a mean of 8.10 ± 3.9 h per day. Demographic and clinical characteristics of the surveyed patients are summarized in Table 1.

**Table 1:** Demographic and clinical characteristics

Variable	Valid N (%)
Disease	
COPD	127 (85.2%)
NMD	8 (5.40%)
OHS	9 (6.00%)
RCWD	4 (2.70%)
Lung fibrosis	1 (0.70%)
Gender	
Male	53 (35.57%)
Female	96 (64.43%)
Age in years (mean ± SD)	69.81 ± 10.15
Smoking	
Current smokers	31 (21.00%)
Previous smokers	93 (62.00%)
Never smoked	25 (17.00%)
Pack-year (mean ± SD)	59.83 ± 43.98
BMI (k/m^2^) (mean ± SD)	30.39 ± 8.64
ED visits in past year per patient due to respiratory problems (mean ± SD)	2.84 ± 2.73
Hospital admission in past year per patient due to respiratory problems (mean ± SD)	2.96 ± 3.00
HMV use in months (mean ± SD)	32.6 ± 36.20
HMV use per day (mean ± SD)	8.1 ± 3.90

*COPD* chronic obstructive pulmonary disease, *NMD* neuromuscular diseases (NMD), *OHS* obesity hypoventilation syndrome, *RWCD* restrictive chest wall disorders, *SD* standard deviation, *HMV* home mechanical ventilation

### Dimensionality and internal construct validity

**Fig. 1:**
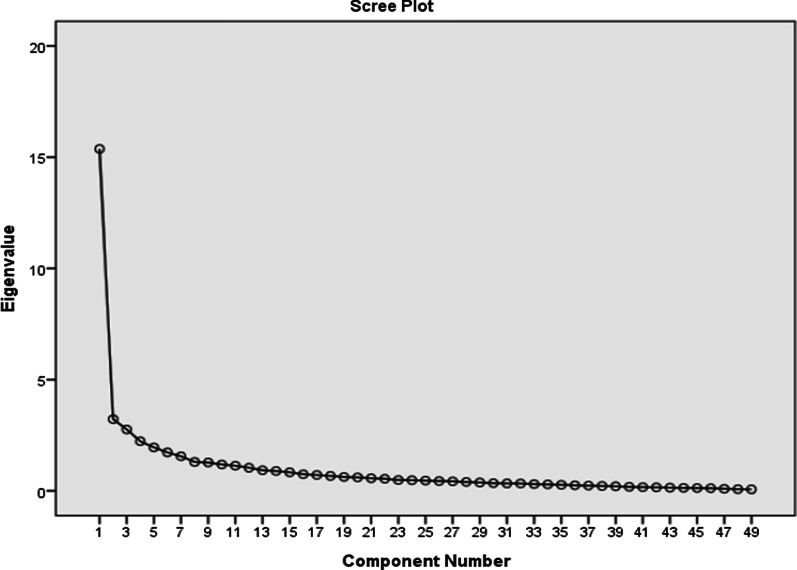
Scree plot using the principal component analysis

The scree test (Fig. 1) suggests that a 1-factor solution is optimal. The ratio of the first to second eigenvalue in our sample was greater than 4, which is further evidence for unidimensionality of the overall scale.

Initial CFA on each subscale did not show a good fit. Modification indices suggested the correlation of residual errors for some items in the SRI-RC, SRI-AS, SRI-WB and SRI-SF sub-scales to improve fit. This resulted in an acceptable RMSEA 90% CI for the subscales RC, WB & SF (upper bound did not exceed 0.1). Moreover, CFI results reflected a good model fit of the observed values in all subscales (above 0.9) (Additional file 2: Table S1).

### Internal consistency reliability

Scores of the Arabic SRI-SS and all subscales are shown in Table 2. Cronbach’s alpha was high for the summary scale, and all subscales ranging between 0.73 and 0.89 (Table 2).

**Table 2:** Arabic SRI Questionnaire reliability

Scale	Number (n)	Mean ± SD scores	Score ranges (min–max)	Cronbach’s alpha α
SRI-RC	8 items	67.5 ± 18.3	21.88–100.00	0.8
SRI-PF	6 items	42.25 ± 23.7	0–100.00	0.79
SRI-AS	7 items	66.82 ± 18.66	17.85–100.00	0.72
SRI-SR	6 items	69.49 ± 20.0	25.00–100.00	0.79
SRI-WB	5 items	59.86 ± 19.96	19.44–100.00	0.82
SRI-AX	9 items	64.73 ± 22.42	10.00–100.00	0.87
SRI-SF	8 items	57.27 ± 19.39	17.85–100.00	0.76
SRI-SS	7 scales	60.98 ± 16.0	24.73–99.5	**0.89**

Bold value is the Cronbach’s alpha for the summary scale

*RC* respiratory complaints, *PF* physical functioning, *AS* Attendant symptoms and sleep, *SR* social relationships, *AX* anxiety, *WB* psychosocial well-being, *SF* social functioning, *SS* summary scale, *SD* standard deviation

### External nomological validity

Table 3 depicts the observed correlations between each of the subscales of the Arabic SRI and subscales of the Arabic SF-36. All correlations in the table are statistically significant at a P-value < 0.01 level. Correlation between the SF-36-MHC and SF-36-PHC with SRI-SS scale was 0.57 and 0.66, respectively. The highest correlations were found between the Physical Functioning and the Social Functioning subscales of both instruments. Correlations were also high between the SRI-WB and SF-36-MH, and the SRI-AX and SF-36-RE. The lowest correlated subscales were the SRI-PF and the SF-36-MH (*r* = 0.21). The correlation between the SRI-Summary Scale and the SF-36-Physical Health Component Summary (r = 0.66) was slightly higher than the correlation between the SRI-Summary Scale and the SF-36-Mental Health Component Summary (*r* = 0.57).

**Table 3:** Correlation matrix between the Arabic SRI and Arabic SF-36

SRI scales	SF-36 Scales									
	PF	RP	BP	GH	VT	SF	RE	MH	MHC	PHC
RC	.40	.40	.33	.33	.40	.43	.38	.33		
PF	**.81**	.60	.19	.34	.36	.6	.40	.21		
AS	.31	.31	.48	.46	.27	.34	.38	.32		
SR	.52	.55	.33	.50	.33	.59	.56	.47		
AX	.40	.54	.25	.49	.41	.48	.61	.45		
WB	.48	.57	.40	.55	.51	.59	.57	.62		
SF	**.71**	.64	.49	.43	.38	**.74**	.56	.35		
SS	.66	.65	.44	.55	.47	.68	.64	.49	0.57	0.66

The SRI scales: *RC* respiratory complaints, *PF* physical functioning, *AS* attendant symptoms and sleep, *SR* social relationships, *AX* anxiety, *WB* psychosocial well-being, *SF* social functioning, and *SS* summary scale

The SF-36 scales: Physical Functioning (PF), Role Physical (RP), Body Pain (BP), General Health (GH), Vitality (VT), Social Functioning (SF), Role Emotional (RE), Mental Health (MH), Physical Health Component (PHC), and Mental Health Component (MHC)

All correlations in the table are statistically significant at a P-value < 0.01 level

Correlations higher than 0.7 are bold

## Discussion

The evidence supporting the benefits of using a disease-specific questionnaire to assess Health-Related Quality of Life (HRQL) in patients with chronic respiratory insufficiency in multiple languages and contexts necessitates the availability of an Arabic-validated SRI. Following a rigorous translation and back-translation process, and pretesting, the final Arabic-SRI scale showed a high internal consistency reliability, and good external nomological validity. Adding to its utility is the fact that formal Arabic was used in the translation (as opposed to the Lebanese dialect), as a result, the instrument can be used in any Arab-speaking population given that formal Arabic is understood by all Arabic-speaking nations. When assessing reliability, all subscales had high internal consistency almost identical to the original version [17], and results were comparable to findings of other validation studies [18–20, 22].

Our estimates of internal consistency (0.897 vs. 0.89), and percent variance explained by the 49-items (63.0% vs. 60.0%) are comparable to the original findings of the German scale. Also corroborating the findings of Windisch et al. [17], we found high correlations between comparable subscales of the SRI and the SF-36. This implying that the Arabic SRI is capable of accurately and consistently measuring certain aspects of the quality of life available in the SF-36, tool typically applied to assess general HRQL. High correlations, as expected and desired, were attained between the physical functioning subscales of both SRI and SF-36, the SRI-WB and the SF-36-MH, as well as the SRI-AX and the SF-36-RE, which is expected and desirable since these subscales measure similar aspects of quality of life. In contrast, subscales measuring different aspects of quality of life such as SRI-RC and SRI-AS had low correlations with SF-36 subscales, as the latter does not explicitly measure respiratory complaints and sleep disturbances.

The low correlations between the subscales of the SRI and the SF-36 measuring different domains ascertains the necessity of having a tool that assesses unique concepts of HRQL related to patients with chronic respiratory failure that are not available in the SF-36 or other tools.

The study findings need to be interpreted considering certain limitations. Tracheostomized patients were not included in this validation study to make it comparable with the original study, keeping in mind that patients receiving mechanical ventilation via tracheostomy tubes have different characteristics than those receiving it non-invasively and thus have implications on their HRQL. However, validating the Arabic SRI among patients receiving HMV via tracheostomy is of a special interest since the prevalence of this group has been significantly increasing over years. Clinical parameters including blood gas values and lung function test were not collected for the current study since the sample was home-based recruited all over Lebanon governorates rather than hospital/clinical based.

Additionally, and unlike the original version that was self-administered, the Arabic version was surveyor-assisted to ensure the best questionnaire administration technique based on our sample characteristics. Assisted administration was adopted to avoid major discrepancies in the mode of administration due to the relatively higher proportion of elderly subjects, and substantial proportion of patients with low education status in our sample. Therefore, assisted administration might have contributed to the low missing item rate in our study, which could have positively influenced the results as other validation studies used self-administered methodology.

## Conclusion

The Arabic SRI version is a valid and reliable tool with high internal consistency reliability and good external nomological validity, comparable to the original German version. The formal Arabic-SRI might be administered across several Arab-speaking populations to allow a valid assessment of quality of life in patients receiving HMV. Such a tool is useful in future epidemiological studies or clinic-based settings to help delineate factors influencing a better quality of life, evaluate interventions and ultimately inform the allocation of resources within the Lebanese context, and possibly other Arab countries.

## Supplementary Information

**Additional file 1.** Questionnaire.

**Additional file 2. Table S1**. Confirmatory Factor Analysis Results.

## Data Availability

The datasets used and/or analyzed during the current study available from the corresponding author on reasonable request. The authors are happy to share the datasets if the manuscript passes the screening phase and reaches preliminary acceptance.
