# Supplementary material for: Validation of the Arabic Severe Respiratory Insufficiency Questionnaire
Source: BMC Pulm Med. 2021 Oct 11;21:315. doi: 10.1186/s12890-021-01644-x (PMC8504101; doi:10.1186/s12890-021-01644-x)
Supplement: Supplementary file 1 — Additional file 1. Questionnaire. [file 12890_2021_1644_MOESM1_ESM.pdf]

Validation of the Arabic Severe Respiratory Insufficiency Questionnaire

Marwan F. Alawieh[a], Rania N. Bzeih†[b], Abla M. Sibai[c], Mohamad F. El-Khatib[a], Lilian A. Ghandour[c]*, Salah M. Zeineldine[d]*

[a] Department of Anesthesiology/ Inhalation Therapy Department - American University of Beirut Medical Center
[b] Nursing Department - American University of Beirut Medical Center
[c] Department of Epidemiology and Population Health, Faculty of Health Sciences American University of Beirut
[d] Internal Medicine - Pulmonary Division American University of Beirut Medical Center

Part I: General Information

**SD1**	Gender	**1**	Male
		**2**	Female
**SD2**	Age	Age at last birthday [__[__] yrs	
		Date of birth (year only) [_[_[_[_]	
**SD3**	What is your highest educational level?	**1**	Illiterate
		**2**	Read and write
		**3**	Elementary
		**4**	Intermediate
		**5**	Secondary
		**6**	University
		**7**	Post graduate
		**8**	Other, specify: -----------------------
**SD4**	What is your current occupational status	**1**	Housewife
		**2**	Retired
		**3**	Not working (looking for a job)
		**4**	Disabled
		**5**	Employee (full time)
		**6**	Employee (part time)
		**7**	Self employed)
		**8**	Other, specify: -----------------------
**SD5**	What is you marital status?	**1**	Single

		2	Married
		3	Widowed
		4	Separated/Divorced
**SD6**	What is your current smoking status?	**0**	None-smoker (never smoked before) **(If selected, skip to SD15)**
		**1**	Ex-smoker (had smoked at least one cigarette/session of water pipe per day for at least six months but had quit).
		**2**	Current smoker (smoke at least one cigarette or one session of water pipe per day).
**SD7**	How many packs of cigarettes do you currently smoke per day?	**0**	Not applicable
		**1**	Pack: ---------------
**SD8**	At which age did you start smoking cigarettes?	**0**	Not applicable
		**1**	**[__[__] yrs**
**SD9**	If you are an ex cigarette smoker, at which age did you stop smoking?	**0**	Not applicable
		**1**	**[__[__] yrs**
**SD10**	If you are an ex cigarette smoker, how many packs did you use to smoke per day?	**0**	Not applicable
		**1**	Pack: ---------------
**SD11**	How many waterpipe sessions do you currently smoke per day?	**0**	Not applicable
		**1**	Sessions: --------------
**SD12**	At which age did you start smoking waterpipe?	**0**	Not applicable
		**1**	**[__[__] yrs**
**SD13**	If you are an ex waterpipe smoker, at which age did you stop smoking?	**0**	Not applicable
		**1**	**[__[__] yrs**
**SD14**	If you are an ex waterpipe smoker, how many sessions did you use to smoker per day?	**0**	Not applicable
		**1**	Sessions: --------------
**SD15**	Who lives with you at the home?	**A**	I live alone<br>□ No = 0 □ Yes = 1
		**B**	My spouse<br>□ No = 0 □ Yes = 1
		**C**	My child/children

			□ No = 0 □ Yes = 1
		D	My daughter-son in law<br>□ No = 0 □ Yes = 1
		E	Other relatives<br>□ No = 0 □ Yes = 1
		F	A maid<br>□ No = 0 □ Yes = 1
		G	A nurse<br>□ No = 0 □ Yes = 1
**SD16**	Who takes care of you at home?<br>**(help you in daily life duties)**	A	Nobody<br>□ No = 0 □ Yes = 1
		B	My spouse<br>□ No = 0 □ Yes = 1
		C	My child/children<br>□ No = 0 □ Yes = 1
		D	My daughter-son in law<br>□ No = 0 □ Yes = 1
		E	Other relatives<br>□ No = 0 □ Yes =
		F	A maid<br>□ No = 0 □ Yes = 1
		G	A nurse<br>□ No = 0 □ Yes = 1
**SD17**	Who of the above most takes care of you?	Specify: ………………………….	
**SD18**	How would you rate your health compared to other people of the same age?	**1**	Much worse
		**2**	Worse
		**3**	Same
		**4**	Better
		**5**	Much better
**SD19**		A	NSSF<br>□ No = 0 □ Yes = 1

	How are you covering the expenses of the therapy? **(the mechanical ventilation machine and supplemental oxygen)**	**B**	Cooperative of Government Employees<br>□ No = 0 □ Yes = 1
		**C**	Army/police<br>□ No = 0 □ Yes = 1
		**D**	Ministry of public health<br>□ No = 0 □ Yes = 1
		**E**	Private insurance<br>□ No = 0 □ Yes = 1
		**F**	Self payer<br>□ No = 0 □ Yes = 1
		**G**	Other

Part II. Medical history

**MH1**	What is the underlying diagnoses leading to your respiratory failure?	**A**	COPD<br>□ No = 0 □ Yes = 1
		**B**	Kyphoscoliosis<br>□ No = 0 □ Yes = 1
		**C**	Neuromuscular disease<br>□ No = 0 □ Yes = 1
		**D**	Obesity-hypoventilation syndrome<br>□ No = 0 □ Yes = 1
		**E**	Other, specify: ---------------------------
**MH2**	What is the main indication for the initiation of home mechanical ventilation?	**A**	Low oxygen saturation even after using supplemental oxygen<br>□ No = 0 □ Yes = 1

		B	High carbon dioxide levels<br>□ No = 0 □ Yes = 1
		C	Dyspnea during night<br>□ No = 0 □ Yes = 1
		D	Apnea during night<br>□ No = 0 □ Yes = 1
		E	Other, specify: ---------------------------
**MH3**	How many times did you visit the emergency room during the previous year because of respiratory problems?	[__ [__] Times	
**MH4**	How many times were you admitted to the hospital during the previous year because of respiratory problems?	[__ [__] Times	
**MH5**	Have you ever been diagnosed with any of these diseases?	A	Hypertension<br>□ No = 0 □ Yes = 1
		B	Diabetes<br>□ No = 0 □ Yes = 1
		C	Renal diseases<br>□ No = 0 □ Yes = 1
		D	Obesity<br>□ No = 0 □ Yes = 1
		E	Sleep apnea<br>□ No = 0 □ Yes = 1

		F	Depression<br>□ No = 0 □ Yes = 1
		G	Other, specify: -----------------------------------
MH6	How do you rate your physical activity?	1	Unrestricted normal activity
		2	Restrictions on strenuous activity, able to perform undemanding tasks
		3	Very restricted everyday activity but capable of self-care
		4	Very restricted everyday activity and incapable of self-care
		5	Confined to bed/armchair, incapable of self-care
MH7	Have you had headaches during the previous week?	0	No
		1	Yes
MH8	Have you had daytime sleepiness during the previous week?	0	No
		1	Yes

Part III. Home Mechanical Ventilation

MV1	When did you start using the therapy at home (what year)?	Year [_[_][_[_]	
MV2	How long have you been using the therapy at home (for how many years)?	[_[_][_[_] years	
MV3	When do you apply the machine?	1	Only during the day
		2	Only during the night
		3	During the day and the night

**MV4**	How many hours do you use the machine **during the day**?	Specify in hours: -------------------
**MV5**	How many hours do you use the machine **during the night**?	Specify in hours: -------------------
