# Supplementary material for: Validation of the Arabic Severe Respiratory Insufficiency Questionnaire
Source: BMC Pulm Med. 2021 Oct 11;21:315. doi: 10.1186/s12890-021-01644-x (PMC8504101; doi:10.1186/s12890-021-01644-x)
Supplement: Supplementary file 2 — Additional file 2. Table S1. Confirmatory Factor Analysis Results. [file 12890_2021_1644_MOESM2_ESM.docx]

**SUPPLEMENTRAY**

**Validation of the Arabic Severe Respiratory Insufficiency Questionnaire**

Marwan F. Alawieh^a^, Rania N. Bzeih**†**^b^, Mohamad F. El-Khatib^a^, Abla M. Sibai^c^, Lilian A. Ghandour^c^*, Salah M. Zeineldine^d^*

^a^ Department of Anesthesiology/ Inhalation Therapy Department - American University of Beirut Medical Center

^b^ Nursing Department - American University of Beirut Medical Center

^c^ Department of Epidemiology and Population Health, Faculty of Health Sciences American University of Beirut

^d^ Internal Medicine - Pulmonary Division American University of Beirut Medical Center

**Table S1. Confirmatory Factor Analysis Results**

| **Scale** | **SRI-RC** | **SRI-PF** | **SRI-AS** | **SRI-SR** | **SRI-WB** | **SRI-AX** | **SRI-SF** |
| --- | --- | --- | --- | --- | --- | --- | --- |
|  | Item | Item | Item | Item | Item | Item | Item |
|  | [SRI02] 0.61 | [SRI01] 0.43 | [SRI06] 0.39 *(0.35)* | [SRI07] 0.62 | [SRI04] 0.59 | [SRI08] 0.63 | [SRI03 0.55 *(0.58)* |
|  | [SRI05] 0.73 *(0.74)* | [SRI16] 0.57 | [SRI09] 0.61 | [SRI10] 0.49 | [SRI20] 0.69 | [SRI13] 0.68 | [SRI15] 0.81 *(0.84)* |
|  | [SRI12] 0.561 | [SRI32] 0.75 | [SRI11] 0.26 *(0.21)* | [SRI21] 0.82 | [SRI30] 0.69 | [SRI26] 0.63 | [SRI23] 0.38 *(0.37)* |
|  | [SRI19] 0.830 | [SRI33] 0.77 | [SRI14] 0.21 *(0.16)* | [SRI27] 0.35 | [SRI34] 0.39 | [SRI28] 0.84 | [SRI31] 0.70 |
|  | [SRI22] 0.568 | [SRI41] 0.51 | [SRI17] 0.84 *(0.82)* | [SRI43] 0.83 | [SRI36] 0.84 | [SRI39] 0.71 | [SRI35] 0.68 |
|  | [SRI24] 0.263 | [SRI45] 0.80 | [SRI18] 0.84 *(0.87)* | [SRI46] 0.53 | [SRI38] 0.80 |  | [SRI37] 0.63 |
|  | [SRI25] 0.298 |  | [SRI42] 0.27 *(0.26)* |  | [SRI40] 0.43 *(0.39)* |  | [SRI47] 0.63 |
|  | [SRI29] 0.678 |  |  |  | [SRI44] 0.65 *(0.66)* |  | [SRI48] -0.05 *(-0.03)* |
|  |  |  |  |  | [SRI49] 0.79 *(0.81)* |  |  |
| **CFI** | 0.78 | 0.91 | 0.68 | 0.90 | 0.72 | 0.93 | 0.87 |
| **CFI after modification** | 0.98 | - | 0.90 | - | 0.97 | - | 0.97 |
| **RMSEA**  **90% CI** | ­­­ RMSEA is 0.16  P-value 0.00  (0.13; 0.19) | RMSEA is 0.13  P-value 0.003  (0.08; 0.18) | RMSEA is 0.21  P-value 0.00  (0.17; 0.25) | RMSEA is 0.13  P-value is 0.02  (0.09; 0.18) | RMSEA is 0.22  P-value is 0.000  (0.198; 0.25) | RMSEA 0.15  P-value 0.005  (0.09; 0.28) | RMSEA is 0.11  P-value 0.001  (0.08; 0.15) |
| **RMSEA**  **90% CI**  **after modification** | 0.04  P-value 0.56  (0.00; 0.08) | - | RMSEA is 0.12  P-value 0.002  (0.08; 0.17) | - | RMSEA is 0.07  P-value is 0.11  (0.03; 0.10) | - | RMSEA is 0.35  P-value 0.001  (0.000; 0.09) |

Italic values represent the factor loading after correlating residual errors of modification indices more than 15.
